# Boerhaave Syndrome: Case Report on a Rare Complication of Semaglutide Use

**DOI:** 10.7759/cureus.117633

**Published:** 2026-10-08

**Authors:** Molly K McMahon, Lauren Smithwick, Andrew Madsen, Ethan Whitt, Blaine Warmbrod

**Affiliations:** 1 Emergency Medicine, The University of Texas at San Antonio Health Science Center, San Antonio, USA; 2 Medicine, The University of Texas at San Antonio Health Science Center, San Antonio, USA

**Keywords:** boerhaave syndrome, endocrinology and diabetes, glp-1, sepsis, shock

## Abstract

Esophageal perforation is a rare but life-threatening condition with high morbidity and mortality, particularly when diagnosis is delayed. Boerhaave syndrome, a spontaneous esophageal rupture often triggered by forceful emesis, can present variably and is frequently difficult to recognize early. We report the case of a 62-year-old female patient who developed Boerhaave syndrome two days after initiating semaglutide therapy for weight loss. She presented with intractable nausea, vomiting, and acute chest pain. The chest CT was concerning for esophageal perforation. She rapidly deteriorated into hypoxemic respiratory failure and septic shock. She underwent emergent thoracotomy with primary repair of the perforation. She continued to be treated for septic shock in the ICU, but ultimately she recovered and was discharged on hospital day 17. As GLP-1 use continues to expand, clinicians should maintain awareness of rare but life-threatening complications such as Boerhaave syndrome, as early recognition, prompt imaging, and timely surgical intervention are critical to improving outcomes.

## Introduction

Boerhaave syndrome, a spontaneous esophageal perforation, most commonly occurs following forceful emesis. Although classically described by Mackler’s triad, namely vomiting, chest pain, and subcutaneous emphysema, these findings are present in fewer than half the patients, contributing to diagnostic difficulty [1]. Esophageal perforation is a rare but life-threatening condition, with an estimated incidence of 3.1 cases per 1,000,000 individuals annually [1]. In one meta-analysis of 944 patients across 46 studies, the immediate mortality rate in patients initiating treatment within 24 hours was 3% with a 95% CI of 0.01-0.08, while in patients whose treatment was delayed beyond 24 hours, the immediate mortality rate rose to 12% (95% CI: 0.08-0.18) [2]. In a recent Boerhaave-specific cohort, disease-specific mortality was 0% among patients diagnosed within 24 hours compared with 38% among those diagnosed after 24 hours (p = 0.042). In this same cohort, seven of the 21 (33%) patients were initially misdiagnosed [3]. After perforation, the absence of a serosal layer in the esophagus facilitates rapid spread of luminal contamination into adjacent mediastinal and pleural spaces, potentially leading to mediastinitis, sepsis, and multiorgan failure. 

Glucagon-like peptide-1 receptor agonists (GLP-1 RAs), including semaglutide, have gained widespread use for weight loss. Recent data indicate a substantial rise in GLP-1 RA prescriptions, with over one million new users in the United States between 2011 and 2023, including a >700% increase among non-diabetic individuals from 2019 to 2023 [4]. While generally well tolerated, these medications are associated with gastrointestinal adverse effects, including nausea, vomiting, and delayed gastric emptying [5]. In a meta-analysis, secondary outcomes showed that the risk of developing gastrointestinal adverse events was 1.59 times higher with semaglutide (risk ratio (RR) 1.59, 95% CI (1.34, 1.88), p<0.00001). Risk for discontinuation due to adverse events was twice as likely in the semaglutide group (RR 2.19, 95% CI (1.36, 3.55), p=0.001) [5]. In the STEP (Semaglutide Treatment Effect in People With Obesity) 5 trial, gastrointestinal adverse events were reported in 82.2% of participants receiving semaglutide and 53.9% receiving placebo and were mostly mild to moderate [6]. As use expands, particularly for off-label use for weight loss management [7], recognition of rare but serious complications becomes increasingly important. We present a case of Boerhaave syndrome occurring shortly after initiation of semaglutide therapy, highlighting a potential association between possible semaglutide-associated vomiting and esophageal rupture.

## Case presentation

A 62-year-old female with a history of thyroid cancer post-resection 10 years prior, on levothyroxine, and on Plaquenil for rheumatoid arthritis was transferred to our facility for suspected Boerhaave syndrome. She reported being in her usual state of health until one day prior to presentation, when she developed intractable nausea and vomiting. She stated she began having nausea and then threw up multiple times, stating the emesis was forceful and non-bloody and non-bilious. She stated that around 5 PM, after multiple rounds of emesis, she felt a “pop” in her chest, and she immediately started having chest pain, prompting activation of emergency medical services. These symptoms began two days after increasing her semaglutide therapy.

At an outside hospital, imaging revealed pneumomediastinum, bilateral pleural effusions (right greater than left), and concern for distal esophageal perforation. She was subsequently transferred for higher-level care. Our facility, a quaternary hospital and level-1 trauma center, was the closest hospital meeting the patient’s specific needs. A CT confirmed pneumomediastinum extending to the lung bases with associated pleural effusions (Figure 1).

**Figure 1:**
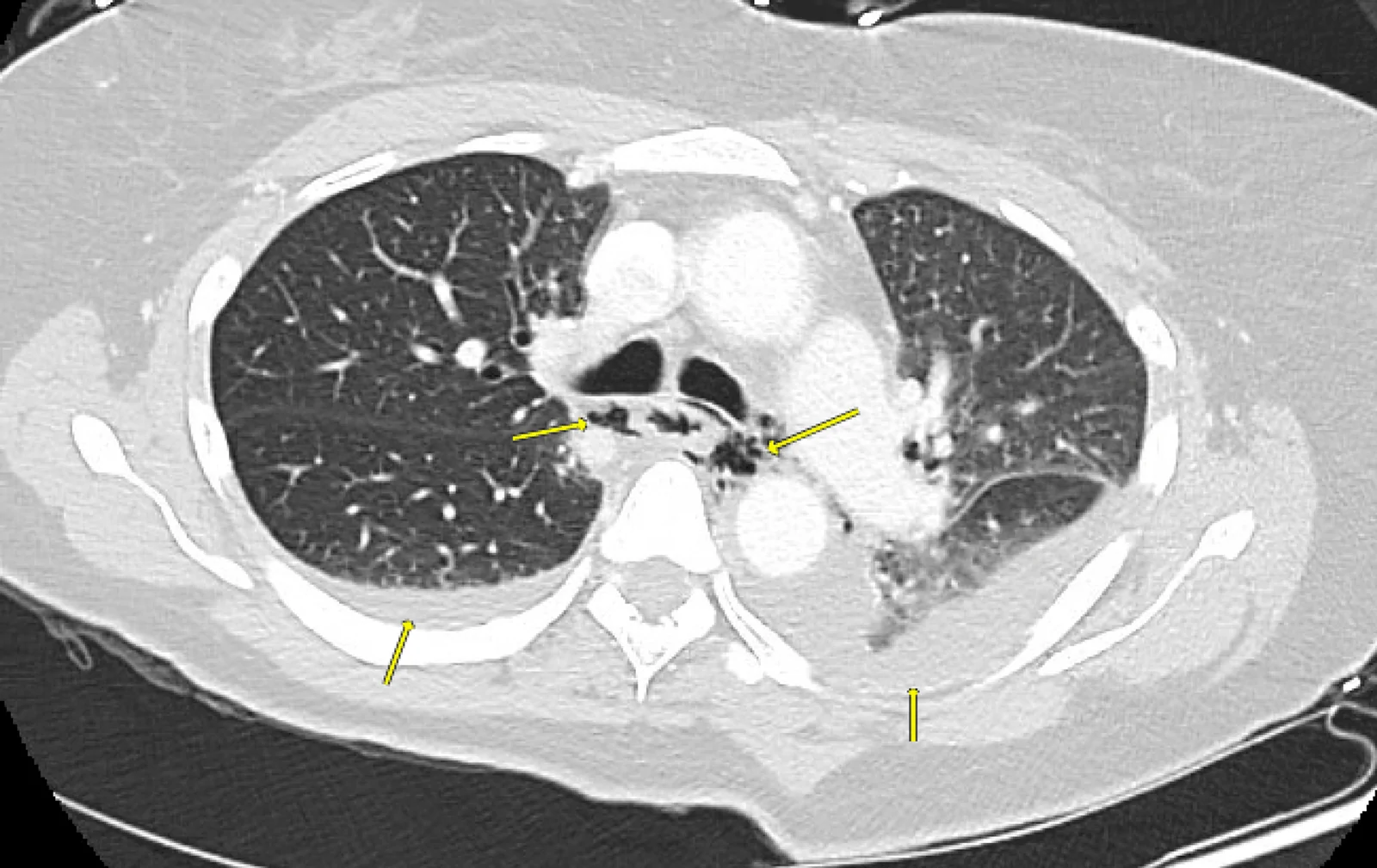
CT confirming pneumomediastinum and bilateral pleural effusions Pneumomediastinum is seen extending to the bilateral neck bases with bilateral small pleural effusions (yellow arrows), left greater than right, and associated right lower lobe atelectasis.

Upon arrival at our facility at 12:50 AM, the patient was tachypneic with a respiratory rate of 30 breaths/min, normal blood pressure of 118/79, a normal heart rate of 63 bpm, and oxygen saturation of 94% on 10 L via an oxymask. She began to experience clinical deterioration at 1:57 AM, with worsening tachypnea with a respiratory rate of 37 breaths/min, hypoxemia at 91% despite being on the 10L oxymaks, and declining blood pressure. Her oxygen was increased to 15 L via an oxymask at 2:15 AM; she acutely decompensated, with oxygen saturation dropping to 89% and blood pressure to 69/36 mmHg, concerning for acute respiratory failure and septic shock. The patient was additionally started on piperacillin-tazobactam.

She underwent emergent intubation for respiratory failure. Prior to intubation, she received sodium bicarbonate for worsening acidosis, with Istat showing a pH of 7.13, carbon dioxide (CO₂) of 69, bicarb of 23, and lactate of 3.6. Additionally, she received a push dose of phenylephrine and initiation of norepinephrine, resulting in transient hemodynamic improvement. Initial X-ray imaging re-demonstrated pneumomediastinum and bilateral pleural effusions (Figure 2). 

**Figure 2:**
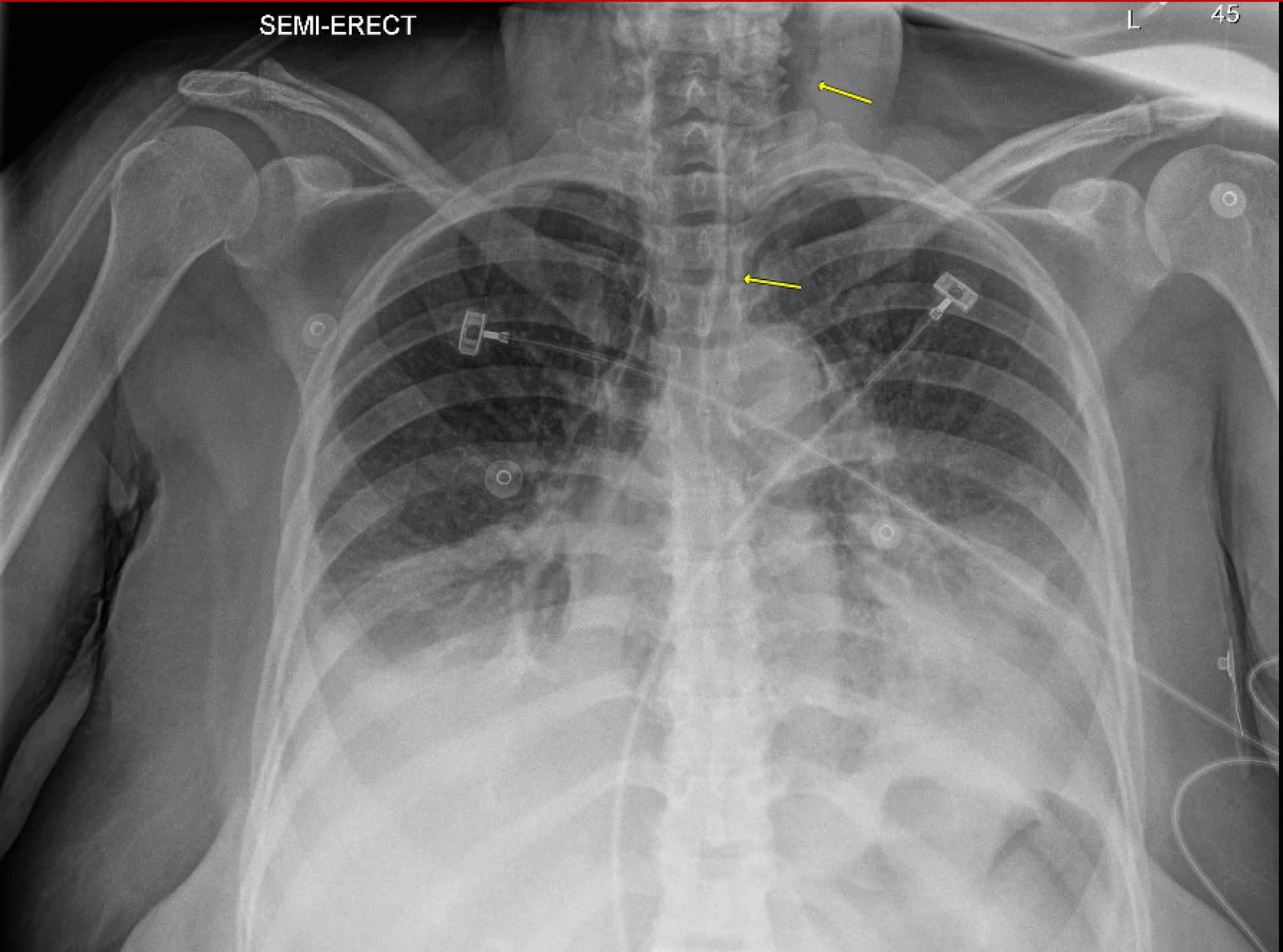
Initial X-ray imaging re-demonstrating pneumomediastinum and bilateral pleural effusions Pneumomediastinum is observed to be extending to the bilateral neck bases (yellow arrows), with bilateral pleural effusions and adjacent atelectasis and/or airspace opacification.

The patient was admitted to cardiothoracic surgery and underwent emergent left thoracotomy, endoscopy, and percutaneous endoscopic gastrostomy placement. Intraoperatively, the patient was found to have a 2 cm full-thickness perforation of the left lateral esophageal wall 37 cm into the esophagus. At this point, to facilitate repair, the cardiothoracic surgeon placed a percutaneous endoscopic gastrostomy tube and proceeded with a left thoracotomy; the perforation was successfully repaired. Intraoperatively, the patient had bilateral pleural effusions, and bilateral chest tubes were placed in the OR. A postoperative chest radiograph on day two demonstrated significant improvement (Figure 3).

**Figure 3:**
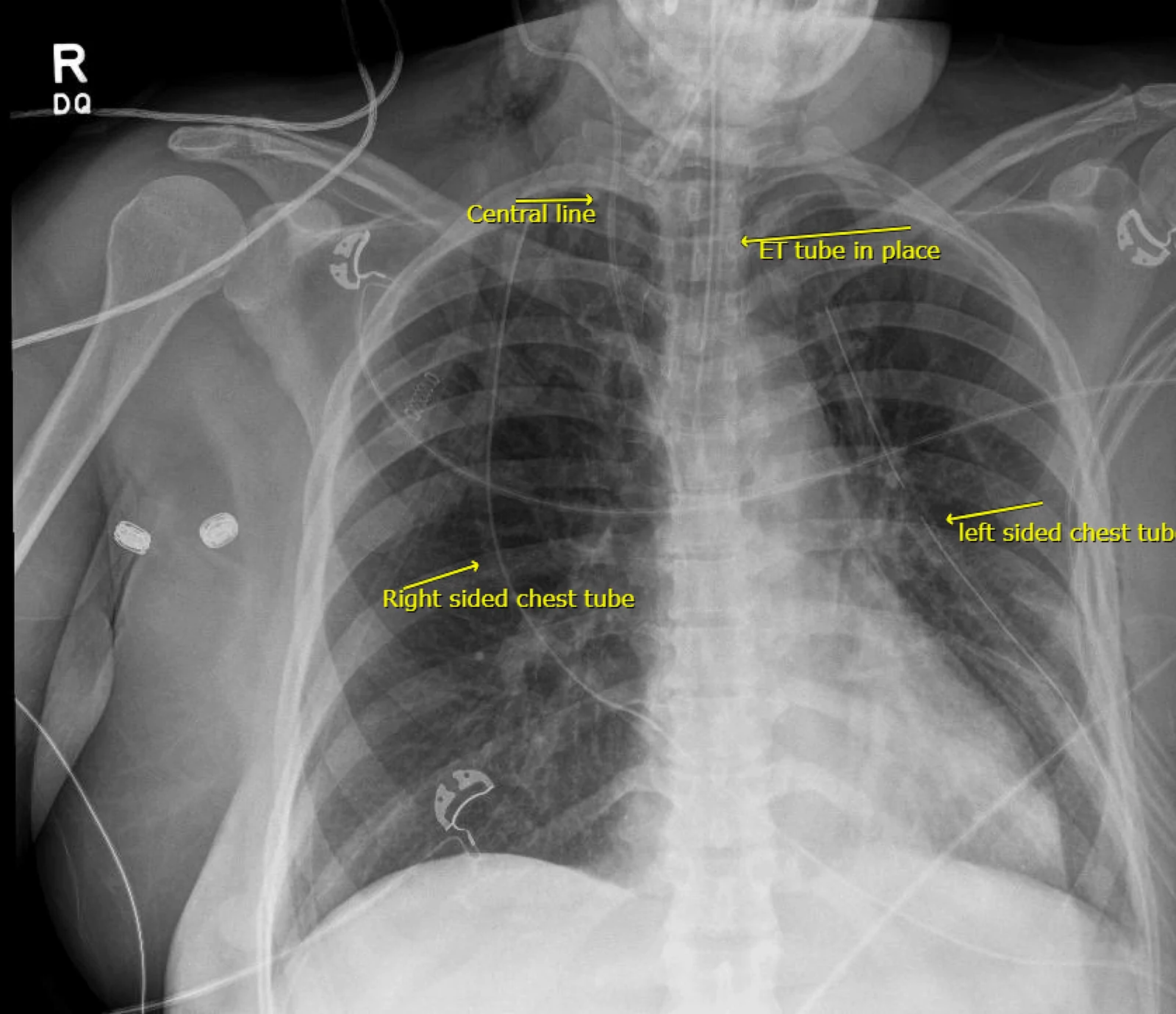
Postoperative X-ray demonstrating almost complete resolution of bilateral pleural effusions, chest tube, and central venous catheter placement Postoperative X-ray with endotracheal (ET) tube and chest tube placement demonstrated improvement in bilateral pleural effusions, persistent left basilar/retrocardiac opacity suggestive of atelectasis, and persistent mild pneumomediastinum.

In the ICU, she continued to require hemodynamic support and was placed on norepinephrine, epinephrine, and vasopressin and received stress-dose steroids. She was found to have Klebsiella bacteremia and was continued on antibiotic therapy. While on vasopressors, she sustained a mean arterial pressure >65 and was slowly weaned off and was off all vasopressors on postoperative day (POD) three. She continued to require mechanical ventilation postoperatively but was ultimately able to be extubated and had her right-sided chest tube removed on POD five. She was downgraded from the ICU on POD six. On POD 10, a CT esophagram and CT chest showed no evidence of an active esophageal leak but worsening pleural effusions and concern for a right-sided loculated effusion (Figures 4-5). Despite appropriate antibiotic therapy, the patient continued to have a fever, with findings of a loculated pleural effusion; a right-sided pigtail was placed (Figure 6). Her chest tubes were removed on hospital day 14, and she was discharged home on a full liquid diet with home health services on hospital day 17. She continued to follow up with cardiothoracic surgery as an outpatient. She was advanced to a soft diet but had to be readmitted to the hospital two months after her initial hospitalization for poor per os (PO) intake and dehydration. She was diagnosed with esophageal dysmotility and esophagitis. She ultimately underwent esophageal stenting. Her PO tolerance improved, and she was able to have her G-tube removed. At the most recent follow-up visit, the patient was doing well. She was eventually restarted on her GLP-1 without recurrent GI symptoms.

**Figure 4:**
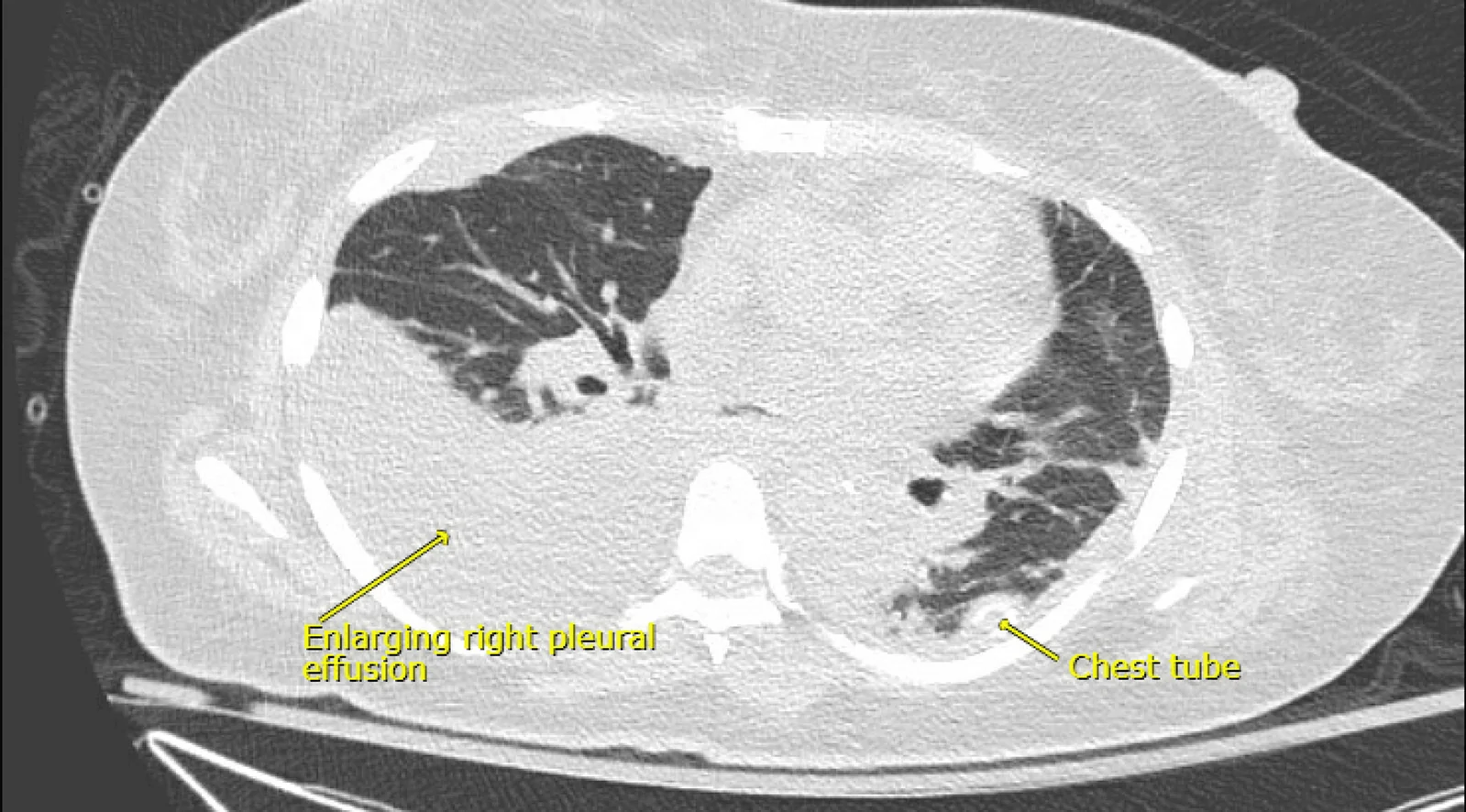
CT esophagram on POD 10 demonstrating enlarging right sided pleural effusion Observed is an increase in loculated moderate-to-large right pleural effusion and right-greater-than-left bilateral lower lobe passive atelectasis secondary to the loculated pleural effusions. POD: Postoperative day

**Figure 5:**
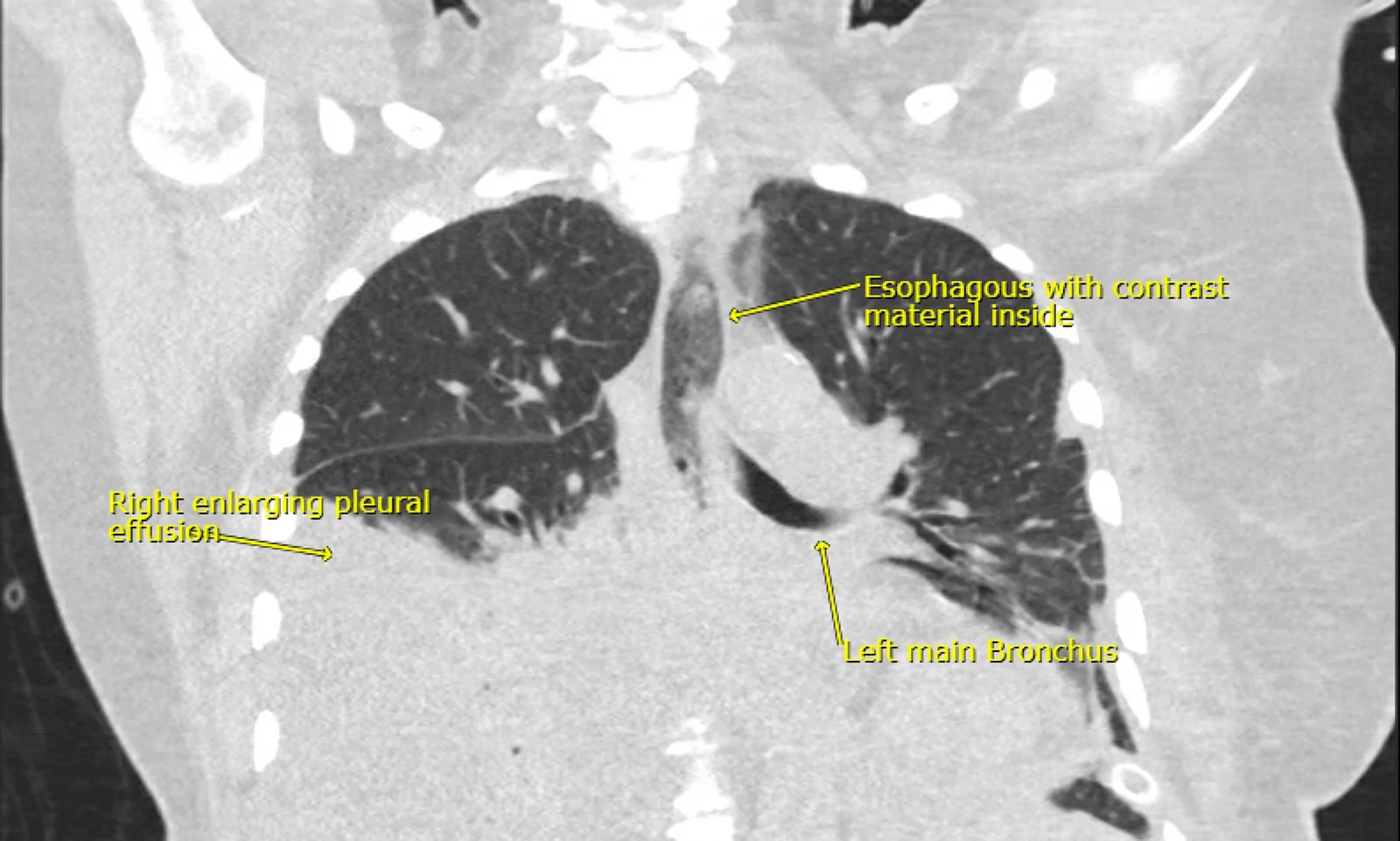
CT esophagram on POD 10 demonstrating no esophageal leak but worsening pleural effusion on the right There is no evidence of extraluminal enteric contrast material to suggest active esophageal leak and interval resolution of previous trace pneumomediastinum. An increase in loculated moderate-to-large right pleural effusion and right-greater-than-left bilateral lower lobe passive atelectasis secondary to the loculated pleural effusions were noted. POD: Postoperative day

**Figure 6:**
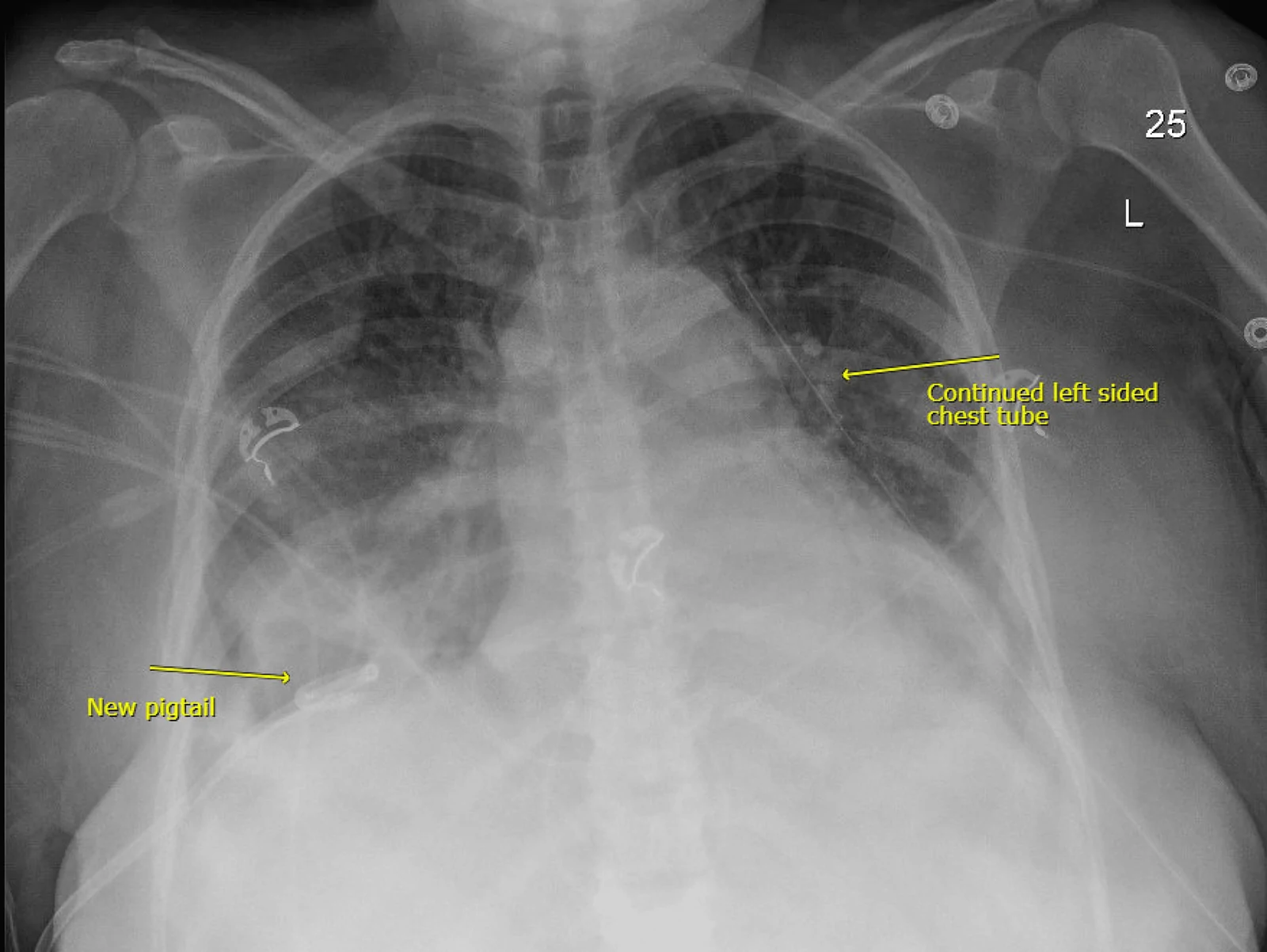
Chest X-ray after placement of right-sided pigtail Seen is the right-sided pigtail placement for loculated right-sided pleural effusion. The patient continued to have a left-sided chest tube that was initially placed intraoperatively.

## Discussion

Esophageal perforation is an uncommon but highly lethal condition that is attributed to a rapid increase in intraesophageal pressure leading to full-thickness tears of the esophagus. Typically, perforations occur longitudinally in the lower one-third of the esophagus. This region is weaker because of a high concentration of neurovascular structures and fewer longitudinal muscle fibers, creating a weak point where most tears occur [8]. Esophageal tears can present with many different symptoms, and clinicians should consider esophageal perforation if a patient presents with neck, chest, or abdominal pain following bouts of emesis. However, these symptoms are not reliable for diagnosing esophageal tears [9]. Thus, clinicians should maintain a high index of suspicion and obtain imaging in patients in whom they are considering esophageal perforation. Plain films can show subcutaneous or mediastinal emphysema, mediastinal widening, and pleural effusions. However, contrast-enhanced CT and CT esophagography are the tests of choice for diagnosing esophageal perforation. Computed tomography is highly sensitive (92% to 100%) in detecting esophageal perforations [9]. Early recognition is critical and depends on clinicians having a high index of suspicion. This case demonstrates how rapidly patients can deteriorate with this condition. The patient in this case developed nausea and vomiting followed by severe chest pain at approximately 5:00 pm. Within several hours, she experienced progressive clinical decline, requiring intubation and vasopressor support by 2:15 AM, ultimately progressing from vomiting and chest pain to septic shock and respiratory failure. 

Severe vomiting occurring shortly after a semaglutide dose increase was considered a possible proximal trigger for the esophageal rupture. Gastrointestinal adverse effects are among the most common side effects associated with GLP-1 RAs, with one study reporting 11.4-20% of patients treated with semaglutide reported nausea and 4.5-11 reporting nausea [10]. Approximately 4% to 8% of participants discontinued therapy because of gastrointestinal intolerance [11]. Nausea, vomiting, and other gastrointestinal symptoms occur more frequently in patients started on higher initial doses or those undergoing rapid dose escalation, with some patients developing symptoms within the first week of therapy [10]. Patients who were started on, for example, 2.5 mg of oral semaglutide had a lower rate of developing nausea (13%) than those started on 10 mg (33%) [12]. Additionally, those who had an eight-week dose escalation reported lower rates of vomiting (16%) than those on a two-week dose escalation (24%) [12]. A separate study showed a similar dose-dependent relationship with both short- and long-acting GLP-1 RAs and found that gastrointestinal symptoms increased further when these agents were used with metformin [13]. Although these adverse effects are typically mild and self-limited, they can occasionally become severe enough to require hospitalization. In the case presented, severe vomiting after semaglutide initiation may have precipitated the esophageal rupture. Similarly, another published case described Boerhaave syndrome occurring after a patient was restarted on a high-dose GLP-1 RAs without repeat titration [14], and a third case described a patient who independently increased her GLP-1 RA and was found to have a very small esophageal perforation after several days of nausea and vomiting [15], further highlighting a possible link between high GLP-1 RAs exposure and severe gastrointestinal symptoms. Although isolated case reports cannot establish causality, the increasing use of GLP-1 RAs should heighten clinicians' awareness of these severe complications that may arise from otherwise expected gastrointestinal side effects. 

This case underscores the importance of including esophageal perforation in the differential diagnosis of patients presenting with chest pain following forceful vomiting, particularly those recently started on GLP-1 RAs. Given the devastating consequences of delayed diagnosis, prompt imaging and early surgical consultation are essential when this condition is suspected. As GLP-1 RAs use continues to expand, awareness of this potential association may facilitate earlier recognition, expedite life-saving intervention, and ultimately improve patient outcomes.

## Conclusions

As GLP-1 use continues to expand for both diabetic and non-diabetic patients, prompt recognition of severe or persistent vomiting and its possible mechanistic consequences is essential. This case highlights how severe gastrointestinal side effects, particularly severe nausea and vomiting, can lead to esophageal rupture and rapid clinical deterioration. Because the presentation of esophageal perforation is often nonspecific, clinicians should consider esophageal perforation in patients with acute chest pain, dyspnea, or systemic deterioration after forceful emesis, including patients who recently initiated or escalated a GLP-1 RA. Early recognition, prompt imaging, and diagnosis are critical to improving outcomes in this high-risk condition. 

Although this single case cannot establish a causal relationship, this report contributes to the growing discussion of GLP-1s and serious gastrointestinal complications and underscores the need for further investigation into their potential role in precipitating Boerhaave syndrome. Limitations of this paper include the single-patient design and the inability to exclude all alternative causes of vomiting or perforation, incomplete assessment of dose-related risk, and limited ability to establish drug causality.
